# Effects of the ephemeral stream on plant species diversity and distribution in an alluvial fan of arid desert region: An application of a low altitude UAV

**DOI:** 10.1371/journal.pone.0212057

**Published:** 2019-02-27

**Authors:** Xiao-Dong Yang, Juan Wang, Ming-Shan Xu, Arshad Ali, Yilu Xu, Dane Lamb, Lu-Chun Duan, Kai-Hong Yan, Sheng-Tian Yang

**Affiliations:** 1 Institute of Resources and Environment Science, Xinjiang University, Urumqi, China; 2 Key Laboratory of Oasis Ecology, Urumqi, China; 3 Global Centre for Environmental Remediation (GCER), The University of Newcastle (UON), Newcastle, Australia; 4 College of Water Science, Beijing Normal University, Beijing, China; 5 Faculty of Geographical Science, Beijing Normal University, Beijing, China; 6 College of Ecological and Environmental Sciences, East China Normal University, Shanghai, China; 7 Spatial Ecology Lab, School of Life Sciences, South China Normal University, Guangzhou, Guangdong, China; Feroze Gandhi Degree College, INDIA

## Abstract

Biodiversity conservation, plant growth and spatial distribution of plant species are the central issues in contemporary community ecology. Ephemeral stream may influence soil properties, which in turn may determine biodiversity and function of an ecosystem in alluvial fan of arid desert region. Ephemeral stream is one of the most common natural disturbances, yet the effects of the ephemeral stream on plant communities in terms of species diversity and plant species distribution remain poorly studied. In this study, the information of species distribution, ephemeral stream beds (‘washes’), and the characteristics of plant growth, *i*.*e*. height, crown area, were interpreted at different heights using the images of low altitude unmanned aerial vehicle (UAV). After that, soil properties such as soil texture (sand, silt and clay), soil water content, *p*H, soil organic matter, soil electric conductivity, soil bulk density and the percentage of gravel content, and their relationships with UAV data were assessed in order to explore the influences of ephemeral stream on species diversity, plant growth characteristics and species distribution in an alluvial fan of arid desert region. The results showed that deep-rooted plants were only distributed in washes whereas shallow-rooted plants were distributed in both washes and the outside of washes (‘non-washes’). Species richness was significantly higher in washes than that in non-washes whereas the opposite pattern was true for abundance. Soil properties, plant height and crown area were higher in washes than that in non-washes. Plant height, crown area and the total number of individual plants increased with increasing wash width and per unit length of stream flow. This study highlights that the coupling factors of ephemeral stream, such as soil erosion, particle transport and sedimentation, can dramatically cause changes in soil properties and total number of individual plants, and hence, can influence species diversity, plant growth characteristics and spatial distribution of plant species in an alluvial fan of arid desert regions.

## Introduction

**Fig 1:**
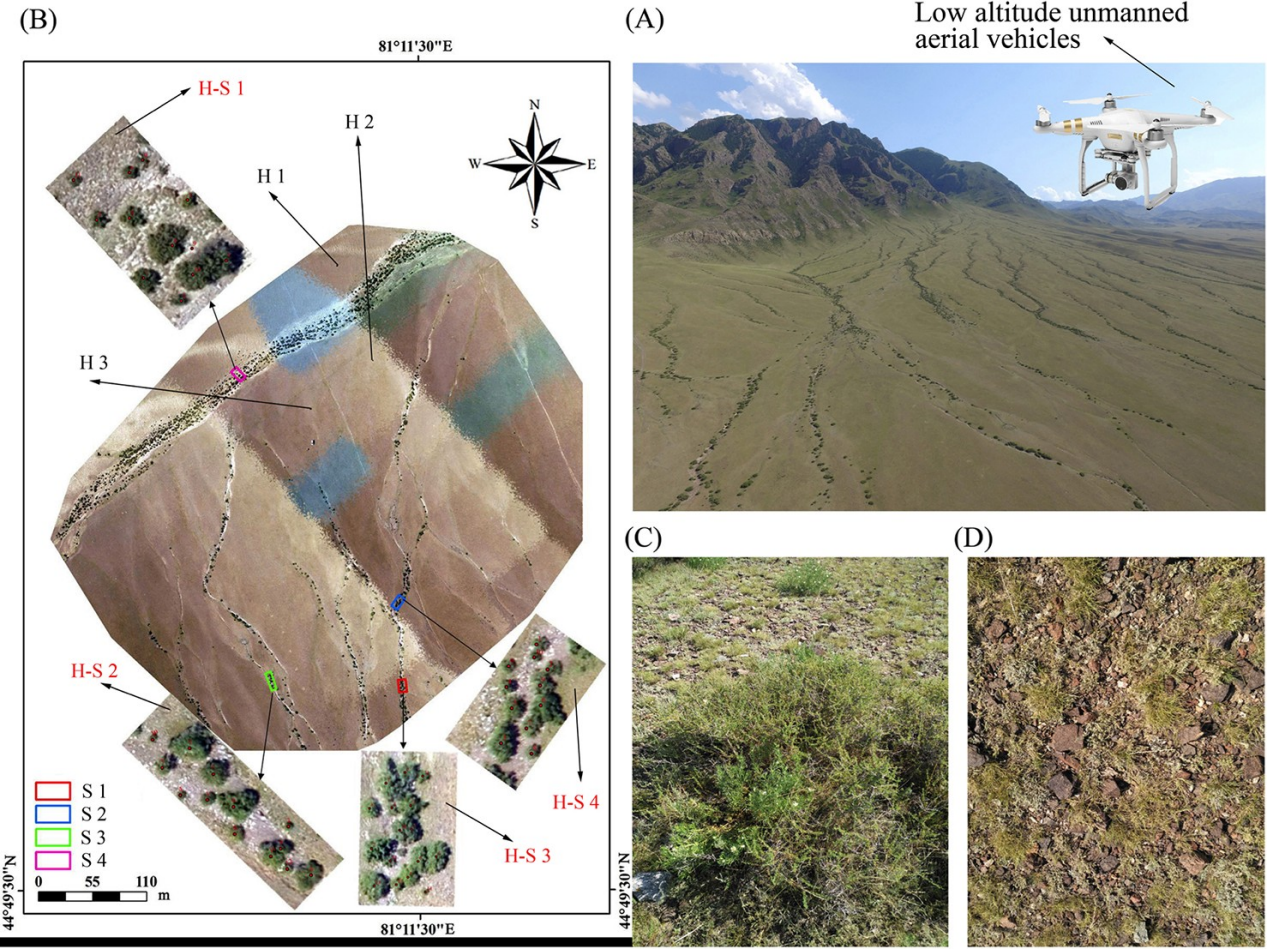
Overview diagrams of experimental site, alluvial fan and species types. A: 200,000 m^2^ (400 m×500 m) sampling area of UAV, which includes four 20 m^2^ shrub and seven 4 m^2^ herbaceous community samples, respectively. SH-1, SH-2, SH-4 and SH-4 refer to herbaceous communities in washes, whereas H1, H2 and H3 are the herbaceous communities in non-washes areas. S1, S2, S3 and S4 are shrub community in washes. B: alluvial fan. C: shrub species. D: herbaceous species. Shrub is *R*. *soongonica*, whereas herbaceous include *H*. *hispidus*, *S*. *kaschgaricum*, *P*. *harmala* and *S*. *capillata*.

An alluvial fan is a triangle-shaped deposit of gravel, sand, and smaller pieces of sediments such as silts, which are usually created as seasonal or intermittent flood interact with mountains, hills, or the steep walls of canyons in arid and semi-arid regions [1]. As a common landscape type in the deserts of arid regions, alluvial fans are widely distributed in the foothills of mountains with elevation ranges from 1500~1900 m [1, 2]. Owing to the strong soil permeability in alluvial fans, water flow from rainfall and glacial melt-water in mountainous areas are usually converted into underground water in arid regions, and eventually can be used for industrial and agricultural ecosystem [2]. The stability of alluvial fans directly relates to water resource security and supply [3], and also regarded as one of the most critical areas of ecosystem conservation in an arid desert region [4, 5]. One of the most prominent surficial features of alluvial fans in arid desert regions is their system of drainage channels, which is easily recognizable in aerial images (Fig 1 and S1 Fig) [6]. Drainage systems caused by the ephemeral stream, are self-organized landscape features that exhibit fractal characteristics of self-similarity across spatial scales [1, 6, 7]. Due to the process of collecting and conducting storm runoff and sediment, the ephemeral stream beds (‘washes’) are hydrologically and biogeochemically distinct from the surrounding soils [6, 8, 9]. However, due to the remoteness of alluvial fans in arid desert regions, the occasional or accidental feature of the ephemeral stream, and both scarcities in quantities of species and plants, the relationships among ephemeral stream, plant species distribution and diversity remain unclear.

Ephemeral stream is one of the most common natural disturbances, and hence can strongly affect plant species diversity, plant growth characteristics and distribution via two main ways in arid desert regions [9–13]. The first reason is soil erosion, which indicates that the undeveloped or shallow-rooted plants may be washed away by the ephemeral stream, thereby the community is mainly constructed from developed-rooted plants in washes [10, 14]. The second reason is soil amelioration, which indicates erosion and collision amongst gravel, and hence soil organic matter and mineral nutrients wrapped in stream can be deposited into soils during the process of channel flow events [7, 12]. Thus, ephemeral stream improves the qualities of soil textural and physicochemical properties in washes of alluvial fans in arid desert regions. In contrast, poor soil properties, a high percentage of gravel and sand contents are common indicators of the outside of washes (‘non-washes’). According to the soil nutrients hypothesis, appropriately increasing soil properties are beneficial to enhance plant adaptation and species diversity [15–17]. In addition, since ephemeral stream as a natural disturbance factor in alluvial fans of arid desert regions, the intermediate disturbance intensity might increase species diversity and other characteristics [6, 9, 18]. Under these expectations, it is possible that ephemeral stream may cause variations in plant growth characteristics, distribution and diversity in alluvial fans of arid desert regions, possibly due to the changes in soil textural and physicochemical properties during channel flow events [6, 8, 9]. Yet, our understandings whether and how ephemeral stream flow affect plant species diversity, growth condition characteristics and speceis distribution in an alluvial fan of arid desert regions remain unclear.

Temporal and spatial investigations of plant communities are the most common methods for assessing biodiversity, plant species distribution and function in both traditional and contemporary community ecology [19, 20]. Under these methods, standard sampling areas are chosen as indicators in order to explain diversity and distribution of plant species [19]. Here, the standard sampling area is determined by vegetation types and sampling positions, *i*.*e*. the minimum sampling area for herbaceous, shrubs, and forest community is 1 m^2^, 25 m^2^ and 400 m^2^ in temperate regions, respectively [21]. However, owing to the limitation of small sampling area and more artificial factors in choosing sampling position, community investigation is regarded as a difficult method to reflect the actual circumstances of plant species distribution and biodiversity [19, 21]. Although large spatial scales and long-term census plots can overcome the above deficiencies of community investigation, it is time-consuming, tedious and labor-intensive in remote geographic areas [22]. In recent years, due to rapid development of unmanned aerial vehicles (UAV), it has become possible to rapidly, flexibly, conveniently and efficiently acquire biophysical characteristics of plant species [20, 23–25]. These advances in UAV can effectively increase the cost-effectiveness of data collection [26, 27]. Therefore, a combination of UAV and field investigation methods can promote the necessary advances in plant species distribution and biodiversity [24–27]. The typical characteristics of an alluvial fan in arid desert region are greater differences in elevation (*i*.*e*. hundreds of meters), steep slopes, and area ranges from 100 to 10,000 m^2^ [2, 7]. Thus, utilization of traditional ecological methods is difficult to cover the whole change caused by flood and its influence on diversity and distribution of plant species in addition to plant growth characteristics.

In this study, the UAV was used at different heights above ground to acquire abundance, species richness, plant height, crown area and wash width. Then, relationships between UAV data and soil properties were used to address the question of whether plant species diversity, distribution and plant growth characteristics differ between washes and non-washes in an alluvial fan of arid desert region. Here, we hypothesized that ephemeral stream would strongly affect diversity and distribution of plant species as well as their growth characteristics due to the changes in the qualities of soil textural and physicochemical properties associated with channel flow events.

## Materials and methods

### Ethics statement

No specific permissions were required for the described field studies. The experimental area is owned and managed by the local government and the location including the site used for our experiment are not privately owned or protected in any way and thus a specific permit for non-profit research is not required. The field studies did not involve endangered or protected plant species in this area.

### Experimental site

The experimental site was conducted on the south slopes of the Tianshan mountains of Wenquan County (E81°11′03″, N44°49′30″) in the northwestern region of Xinjiang Uygur Autonomous Region, China (Fig 1A). The study region is the part of Bortala River basin having an altitude of 1556 m and characterized by an arid desert climate. Based on the observational data of local hydrologic station, the annual rainfall changes greatly with altitude. For example, 400~600 mm rainfall occurs in areas having an average altitude > 1000 m above sea level, whereas 150 mm rainfall occurs at low altitude areas. Rainfall is variable throughout the year, *i*.*e*., spring, summer, autumn and winter account for 31.4, 41.0, 16.5 and 8.1% of the total annual rainfall, respectively. An annual mean potential evaporation is 2281 mm. In this region, the mean temperature in the warmest month (July) is 25.0°C and the mean temperature in the coldest month (January) is -13.4°C.

### Experimental design and measurements

In this study, the selected area of the alluvial fan is approximately 10 ha based on the estimation by Google Earth. Within the selected area, a 200,000 m^2^ (400 m × 500 m) area was randomly selected as the experimental site. The UAV system, which included a UAV (Phantom II, DJI-Innovations Inc., Shenzhen, China), a Cardan suspension and a FC300X_ 3.6_4000x3000 (RGB) action-camera, was employed to acquire high resolution stereoscopic images and topographic data. In this study, based on UAV images, the channel cross-sections were calculated using 3D analyst module of ArcGIS 10.4. The stereoscopic images were processed by the rapid and automatic professional processing software named Pix4Dmapper (https://pix4d.com/). Image treatment includes data importing, initial processing, point cloud encryption, digital orthophoto map (DOM) generation, and digital surface model (DSM) generation [28, 29]. Subsequently, the DEM data was extracted by DSM using interface description language.

The precision of UAV topographic surveys can reach centimeter levels [30, 31], and hence, UAV data can accurately obtain surface features such as height, width, length, area and volume [23, 29]. However, the sampling height of UAV has a negative relation with the identification of species type [30], and, therefore, diversity and spatial distribution of plant species were calculated by the number of plants and species type. Thus, it was necessary for the exploration of biodiversity and plant species distribution to take UAV images at different heights in order to accurately obtain data on species type. Here, 20 m and 150 m were set as the sampling heights to obtain UAV images of herbaceous plants and experimental site, respectively. Image sizes of 20 m and 150 m sampling heights were 20 m^2^ and 200,000 m^2^, respectively. Specifically, 200,000 m^2^ UAV image was first sampled (Fig 1), and then, based on this image, the alluvial fan was divided into two landscape types, *i*.*e*. washes and non-washes. Within both areas, three and four 20 m^2^ images were randomly taken 20 m height, respectively. Generally, diversity and spatial distribution of herbaceous and shrubs communities can be respectively calculated in 1~4 m^2^ and 16~25 m^2^ in an arid desert region [32]. In the study area, shrubs and herbaceous plants were mainly distributed in both washes and non-washes in an alluvial fan of arid region (Fig 1). Thus, a 4 m^2^ herbaceous community plot was randomly resampled in every 20 m^2^ washes and non-washes (see Fig 1; H1, H2, and H3 were resampled in non-washes, whereas H-S1, H-S2, H-S3 and H-S4 in washes). Within each sampled plot, based on DEM and DSM data, wash width, the numbers of individual plants, plant height and crown area were calculated using ArcGIS 10.4. Species types of herbaceous and shrub communities were measured at 4 m^2^ and 20 m^2^ by using visual interpretation, respectively (Fig 1, S1 Table). In many previous remote sensing studies, it may be crucial to test the accuracy of the identification by comparing results from remote sensing and ground-based investigation. However, in this study, because 20 m was set as a sampling height of plants, our obtained UAV image was a high resolution image in centimeter-level. These could be used efficiently to get the precise result of plant growth. In this case, the sampling results of UAV image interpretation should be identical to the ground-based investigation. Hence, we did not compare the remote sensing and ground-based investigation results.

After UAV sampling, three soil samples (0–20 cm depth) in every 4 m^2^ plots were collected randomly and then transported back to the Soil Science Laboratory at Xinjiang University for the determination of soil properties. In this study, soil properties included soil texture (sand, silt and clay, expressed in %), soil water content (%), *p*H, soil organic matter (mg·kg^-1^), soil electric conductivity (mS·cm^-1^), soil bulk density (kg·m^-3^) and gravel content (mass percent; %). Soil electric conductivity and *p*H were determined at 1:2.5 soil-water ratios using a glass electrode and conductivity bridge, respectively [33]. Soil water content and bulk density were measured following the drying and cutting ring methods, respectively [34]. Soil organic matter was measured by Mo-Sb colorimetry, soil texture and the percentage of gravel content were tested using pipette and sieve analysis [34].

### Data analyses

In this study, species diversity was quantified by abundance and species richness. Due to variation in the areas of visual interpretation between herbaceous and shrub communities, the differences in abundance and species richness between washes and non-washes were compared in 4 m^2^ and 20 m^2^ areas, respectively. Here, 4 m^2^ and 20 m^2^ were sampling areas of herbaceous and shrub communities (Fig 1C). Generally, plant height and crown area can be used as indicators of plant growth characteristics [35]. Hence, the differences in both indicators between washes and non-washes can reflect the effects of the ephemeral stream on plant growth. Thus, the comparison of herbaceous and shrub communities between washes and non-washes were also calculated in 4 m^2^ and 20 m^2^ areas, respectively. It is theoretically plausible that the qualities of soil properties increase with the increase in wash width during the process of infiltration [12]. In addition, plants can grow more efficiently when there is an improvement in the soil properties [15, 16]. Therefore, relationships of wash width with tree height and crown area were assessed in order to explore the effects of the ephemeral stream on plant productivity and structural diversity.

Spatial distribution of plant species is the arrangement of plants on earth surface. For a given species, the total number of individuals, plant height and crown area have the obvious relationships with the degree of aggregated distribution [36]. In the study area, there were only one shrub species which grew in the studied washes. Thus, relationships of wash width with the total number of individuals, plant height and crown area can provide an indication of the effect of the ephemeral stream on plant distribution. In this study, the measured unit was a small grid with a length of 2 m. The width was the actual width of washes. Length and width were quantified based on the directions of stream flow and its perpendicular, respectively.

An independent sample t-test was used to examine the differences in diversity (*i*.*e*. abundance and species richness), and soil properties between washes and non-washes. Linear regression was used to measure the relationship of wash width against plant height and crown area, as well as against the total of the number of individuals, plant height and crown area per unit of stream flow length. All statistical analyses were considered to be significant at *P* < 0.05. All statistical analysis was performed using the R version 3.2.2.

## Results

### Differences in species diversity and plant growth characteristics between washes and non-washes

There were only five observed plant species in the studied area of an alluvial fan in arid region. Among them, *Reaumuria soongonica* was the shrub species whereas remaning four were herbaceous species, *i*.*e*., *Heteropappus hispidus*, *Seriphidium kaschgaricum*, *Peganum harmala* and *Stipa capillata*. Based on the results of UAV images and field investigation, our result showed that *H*. *hispidus* and *P*. *harmala* were distributed in washes only, whereas *S*. *kaschgaricum* and *S*. *capillata* were distributed in both washes and non-washes (Fig 1).

**Fig 2:**
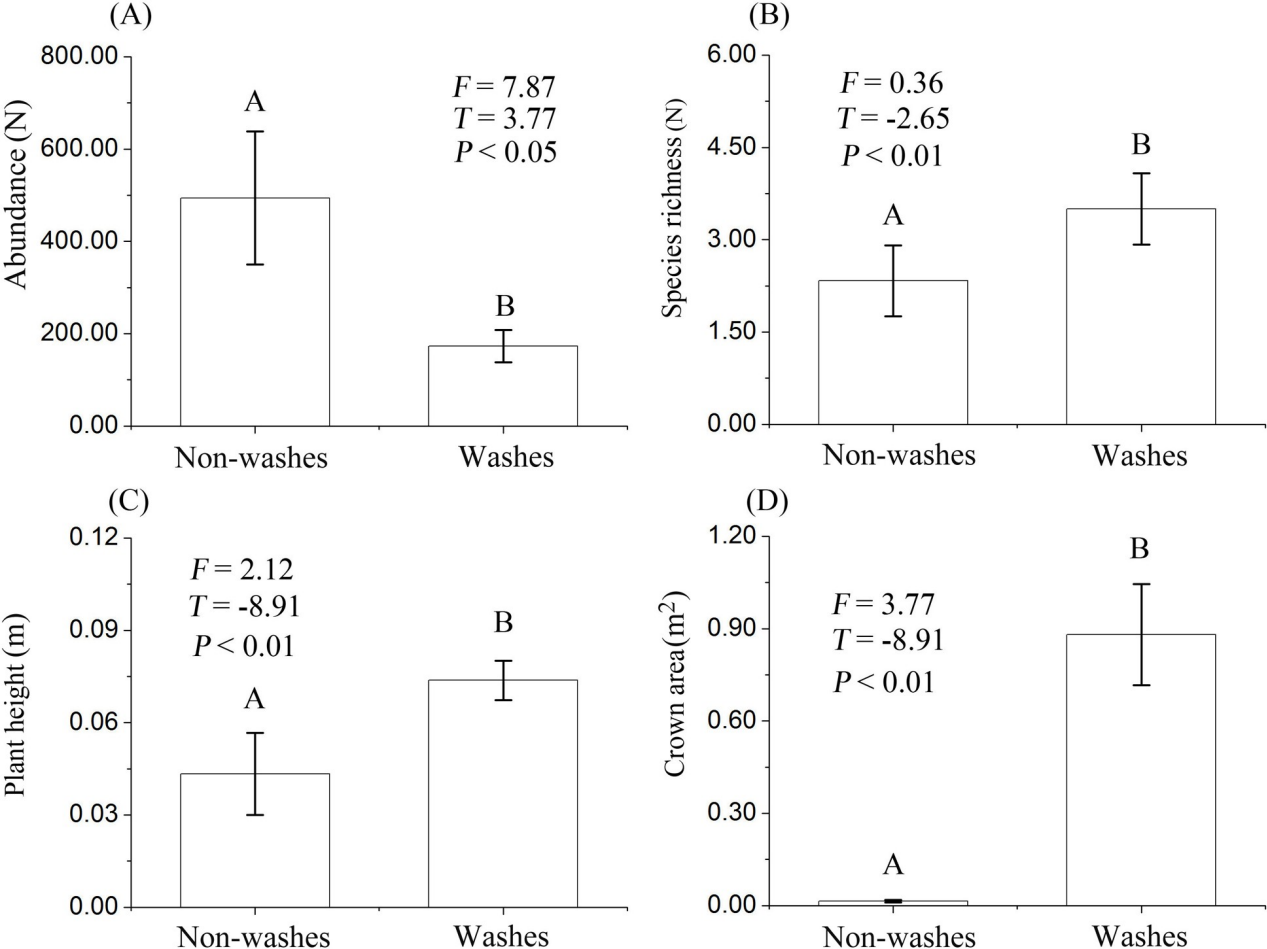
Differences in abundance, species richness, plant height and crown area between washes and non-washes. Different letters in the horizontal direction in each box indicate the significant differences in related variables between washes and non-washes, whereas the same letters show the insignificant difference. Values are shown as *Mean*±*SE*.

In herbaceous community (4 m^2^), species richness, plant height and crown area were significantly higher in washes compared to non-washes (*P*<0.05), whereas abundance showed a contrasting pattern (*P*<0.05) (Table 1). With respect to shrub community (20 m^2^), our results showed also that species richness, plant height and crown area were significantly higher in washes (*P*<0.05). Abundance was lower in non-washes than that in washes (*P*<0.05) (Fig 2).

**Table 1:** Summary of the descriptive statistics for the variables used in this study. Wash width (m) and shrub growth characteristics are interpreted by 200, 000 m^2^, whereas diversity and herbaceous plants growth are calculated in 4 m^2^ UAV images. Plant growth characteristics include plant height (m) and crown area (m^2^). Descriptive statistics include *Mean* ± *SD*, maximum and minimum. *F*, *T*, and *P*-value are the results from independent sample t-test for the differences in diversity and plant growth condition between washes and non-washes. Different letters in the vertical direction with related variables indicate the significant differences between washes and non-washes, whereas the same letters show the insignificant difference.

Types	Sampling position	Sampling area (m^2^)	Variables	Mean±SD	Maximum	Minimum	*F*	*T*	*P*-value
**Washes**		200,000	Width	12.85±10.46	41.60	0.96			
**Shrub in washes**		200000	Height	0.57±0.20	0.95	0.10			
			Crown area	1.63±0.55	3.41	0.35			
**Diversity**	Herbaceous community in non-washes	4	Abundance	79.00±23.06**A**	97.00	53.00	**2.73**	**1.67**	**<0.05**
			Richness	2.33±0.58**A**	3.00	2.00	**0.34**	**-2.64**	**<0.05**
	Herbaceous community in washes	4	Abundance	57.25±11.32**A**	70.00	45.00			
			Richness	3.50±0.58**B**	4.00	3.00			
Plant growth characteristics	Herbaceous community in non-washes	4	Height	0.04±0.01**A**	0.06	0.03	**6.45**	**-3.83**	**<0.05**
			Crown area	0.17±0.03**A**	0.19	0.14	**4.01**	**-9.11**	**<0.01**
	Herbaceous community in washes	4	Height	0.42±0.17**B**	0.61	0.22			
			Crown area	1.08±0.17**B**	1.33	0.98			

### Differences in soil properties between washes and non-washes

Results showed that gravel content, soil bulk density, and *p*H were significantly higher in non-washes than that in washes (*P*<0.05), whereas soil organic carbon and soil water content showed the opposite pattern (*P*<0.05). Soil electric conductivity was not different between washes and non-washes (*P*>0.05) (Table 2). For soil texture, the percentage of sand was higher in non-washes (*P*<0.05), whereas silt and clay showed the opposite pattern (*P*<0.05) (Table 2).

**Table 2:** Differences in soil properties between washes and non-washes. Soil properties includes texture (*i*.*e*. sand, silt and clay) (%), the percentage of gravel content (%), soil bulk density (kg·m^-3^), soil organic carbon (g·kg^-1^), pH, soil electric conductivity (dS·m^-1^) and soil water content (%). *F*, *T*, and *P*-value are the statistical results of the independent sample t-test. Different letters in the horizontal direction with related variables indicate the significant differences in soil properties between washes and non-washes, whereas the same letters show the insignificant difference. Values are represented as *Mean* ± *SD*.

Soil properties	Non-washes	Washes	*F*	*T*	*P*-value
**Sand**	47.33±8.66A	33.67±6.32B	0.15	3.82	**<0.01**
**Silt**	36.33±6.30A	45.00±4.90B	0.14	-3.26	**<0.01**
**Clay**	16.33±4.41A	21.33±5.22B	0.16	-2.19	**<0.05**
**The percentage of gravel content**	9.67±1.66A	7.33±1.58B	0.16	3.05	**<0.01**
**Soil bulk density**	1.38±0.03A	1.27±0.06B	3.57	5.22	**<0.01**
**Soil organic carbon**	0.77±0.23A	2.31±1.54B	8.74	-2.97	**<0.05**
**pH**	8.01±0.20A	7.67±0.16B	0.31	3.96	**<0.01**
**Soil electric conductivity**	1.83±0.11A	1.84±0.12A	0.27	-0.22	0.83
**Soil water content**	3.46±1.01A	6.26±1.27B	3.14	-5.36	**<0.01**

With respect to the different width positions of washes, our results showed that soil organic carbon was significantly higher in wider than in narrower positions (*P*<0.05). Soil gravel content, soil bulk density and *p*H were significantly lower in wider position (*P*<0.05), but soil electric conductivity and soil water content showed no differences (*P*>0.05). For soil texture, the percentage of clay was significantly higher in wider positions (*P*<0.05), whereas sand and silt showed no differences (*P*>0.05) (Table 3).

**Table 3:** Differences in soil properties between narrower and wider positions of washes. Soil properties include texture (*i*.*e*. sand, silt and clay) (%), the percentage of gravel content (%), soil bulk density (kg·m^-3^), soil organic carbon (g·kg^-1^), pH, soil electric conductivity (dS·m^-1^) and soil water content (%). *F*, *T*, and *P*-value are the statistical results of the independent sample t-test. Different letters in the horizontal direction with related variables indicate the significant differences in soil properties between narrower and wider positions of washes, whereas the same letters show the insignificant difference. Values are represented as *Mean* ± *SD*.

Soil properties	Wider position	Narrower position	*F*	*T*	*P*-value
**Sand**	28.33±2.51	36.33±5.98	3.47A	-2.16	0.07
**Silt**	44.33±4.16	45.33±5.57	0.59A	-0.27	0.79
**Clay**	27.33±4.04A	18.33±2.16	1.46B	4.50	**<0.05**
**The percentage of gravel content**	5.67±0.57A	8.17±1.17	1.28B	-3.42	**<0.05**
**Soil bulk density**	1.21±0.03A	1.31±0.03	0.09B	-4.11	**<0.01**
**Soil organic carbon**	4.01±1.46A	1.47±0.61	5.48B	3.81	**<0.01**
**pH**	7.50±0.09A	7.76±0.11	0.27B	-3.39	**<0.05**
**Soil electric conductivity**	1.82±0.14A	1.85±0.13	0.10A	-0.23	0.82
**Soil water content**	28.33±2.51	36.33±5.98	3.47A	-2.16	0.07

### Relationships of wash width with species diversity and plant growth characteristics

**Fig 3:**
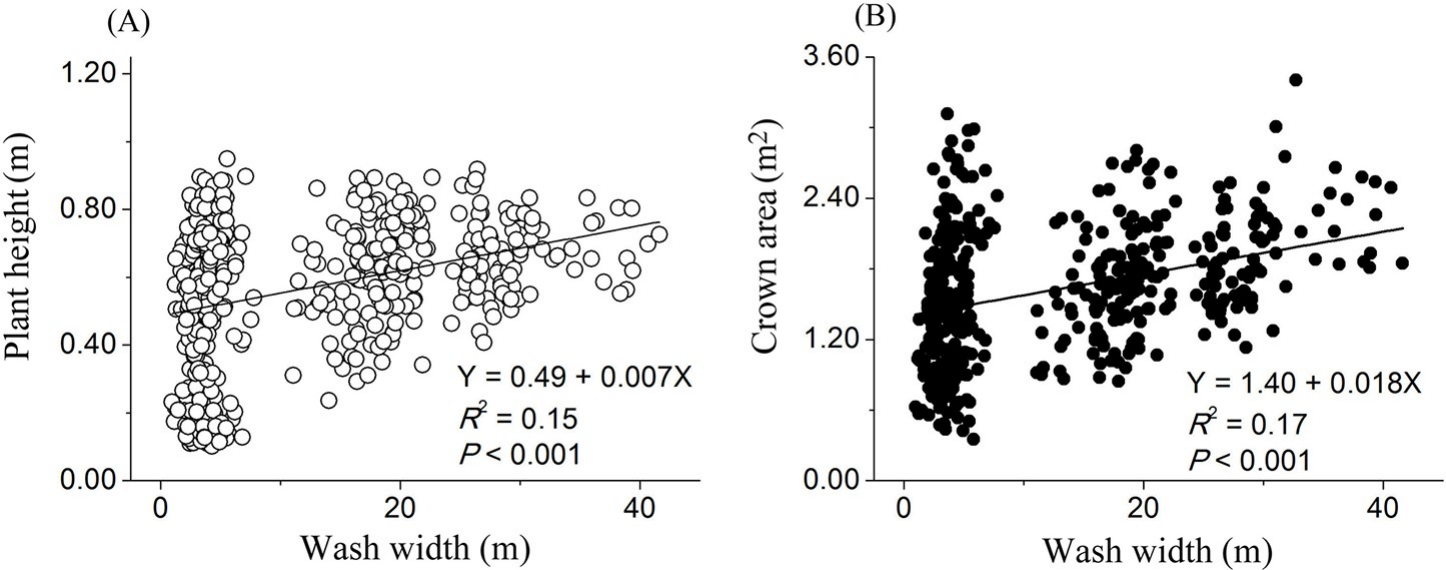
Linear regression relationships of wash width of the ephemeral stream against plant height and crown area. Regression equation, *R*^2^ and *P*-value are presented.

**Fig 4:**
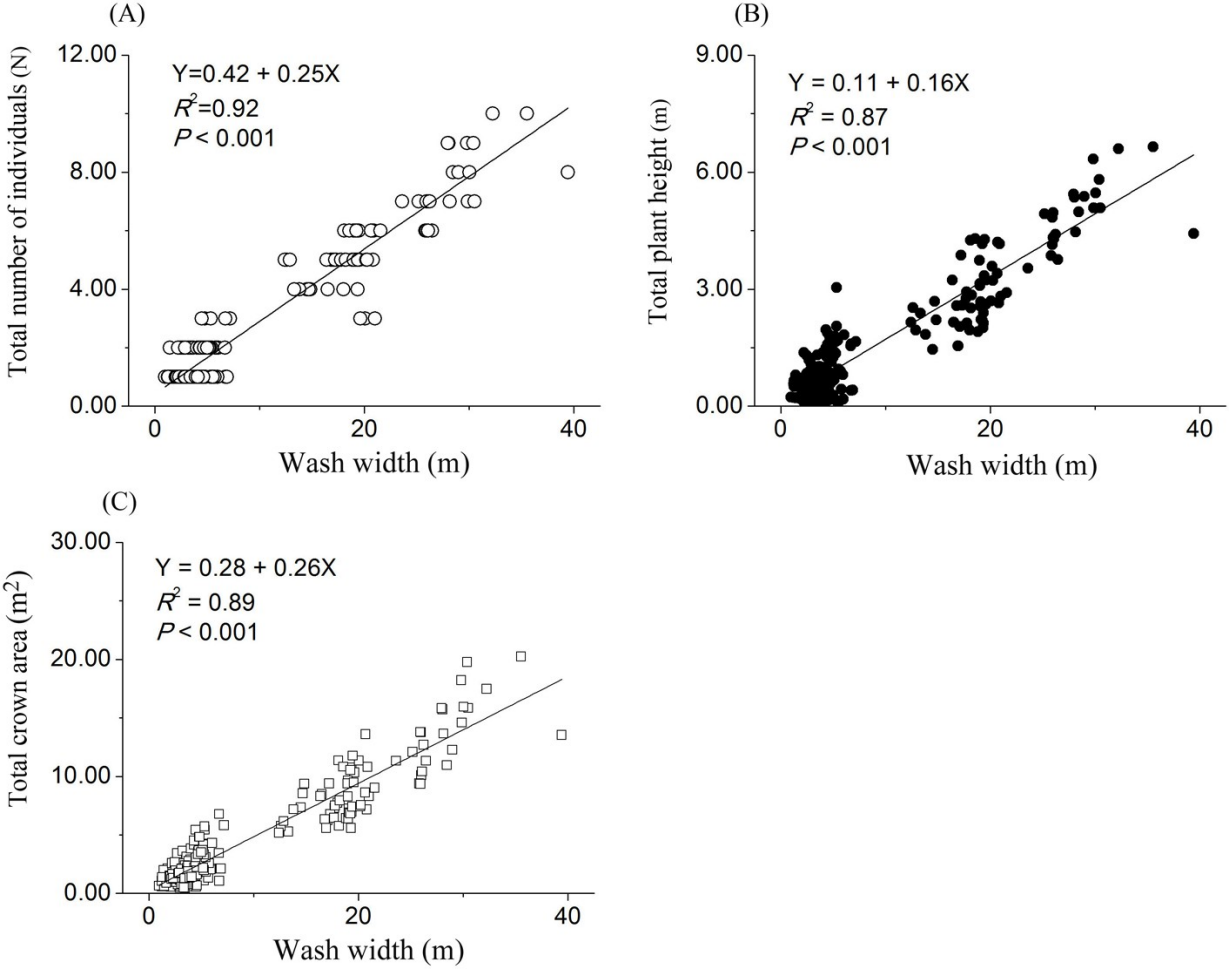
Linear regression relationships of wash width of the ephemeral stream against the total number of individual plants, plant height and crown area per unit of washes. Unit is the small grid with the length as 2 m, and the width as the actual wash width of the ephemeral stream. Length and width are located on the directions of stream flow and its perpendicular, respectively. Regression equation, *R*^2^ and *P*-value are presented.

The simple linear regressions analyses showed that plant height (*R*^2^ = 0.15, *P*<0.001) and crown area (*R*^2^ = 0.17, *P*<0.001) were significantly increased with increasing wash width (Fig 3). Similarly, the wash width per length of washes showed significant linear relationships with the total number of individuals (*R*^2^ = 0.92, *P*<0.001), plant height (*R*^2^ = 0.87, *P*<0.001) and crown area (*R*^2^ = 0.89, *P*<0.001) (Fig 4).

## Discussion

### Effects of the ephemeral stream on species diversity and plant growth characteristics

Ephemeral stream is one of the most common natural disturbances, but the effects of the ephemeral stream on plant communities in terms of species diversity and plant species distribution remains poorly studied [10, 11, 14]. In humid and semi-humid regions, it has been reported that stream has degraded soil properties, species diversity and ecosystem stability, which resulted in stunted growth and sometimes mortality of the plants due to erosion and waterlogging [10]. In contrast, alluvial fans with poor soil characteristics in arid desert regions [2, 3], soil erosion and waterlogging are advantageous in order to decrease the percentage of gravel and sand content in washes and, concomitantly, increase the percentage of clay and silt [2, 6, 8, 9]. In addition, the majority of the ephemeral stream in alluvial fans infiltrated and shifted into groundwater due to the excellent soil permeability [3, 7]. Within this process, soil organic matter and mineral nutrients in streams are continuously deposited into soils, which, in turn, increase the contents of soil organic matter and nutrients while decreasing soil bulk density [6, 8, 9, 14]. Consequently, we showed that the qualities of soil properties in washes were superior to non-washes (Table 2). Previous studies reported that appropriately increasing soil properties, including soil organic matter, parent materials and texture, can enhance the adaptation of majority of plant species in the desert regions [6, 8, 9, 15, 16], thereby the improvement in soil properties has a positive relationship with species diversity [13, 17]. Hence, our results showed that species richness in washes was significantly higher than that in non-washes. These results also showed that the improved soil properties are crucial to understand the variations in plant responses and vegetation structure in desert environments [8, 9].

In addition, the effect of the ephemeral stream on species diversity can also be quantified in term of soil erosion [10, 14]. For example, most of shallow-rooted plants may wash away by stream flow, whereas deep-rooted plants can survive during channel flow events. In this case, the number of individuals would significantly decrease, whereas species richness may or may not dramatically change after a channel flow event [10, 14]. Although washes have the better soil quality than non-washes, soil erosion due to channel flowing may decrease the number of shallow-rooted plants, and subsequently may cause a decline in the total number of individuals in washes as compared to non-washes. Hence, our results support these speculations that abundance in washes is significantly higher than those in non-washes. These observations are consistent with the predictions of the intermediate disturbance hypothesis [6, 18]. Because the channel flowing of the ephemeral stream is a disturbed force of ecosystem in alluvial fans of arid desert region [1, 6, 8, 9], intermediate disturbance facilitates species richness and plant growth condition, whereas decreases abundance and plant spatial distribution.

We also found that plant height and crown area in washes were higher than those in non-washes, suggesting that plant productivity increases following intermediate disturbance. As mentioned above, in alluvial fan of an arid desert region, the ephemeral stream is advantageous to improve soil properties through the reduction in soil *p*H, soil bulk density and the percentages of gravel and sand percentages [6, 8, 9]. Ephemeral stream also provides an improvement in the content of soil organic matter, soil water, and the percentages of clay and silt [6, 9]. Previous studies indicated that the improvement in soil properties is beneficial for plants to capture and absorb water and nutrients, thereby providing better conditions to support plant growth [15–17, 37]. In this study, these mechanisms can be evidenced by the significant relationships of wash width with plant height and crown area. Owing to the influences of ephemeral stream on the improvement of soil properties [2, 14], wash width had a positive relationship with the qualities of soil properties, and hence, plant growth was higher in wider washes. These results are consistent with previous studies in alluvial fans of Mojave Desert, indicated that the higher plant growth condition in washes rather than these in non-washes probably due to the two follow mechanisms [6, 8, 9]: first, infiltration occurring during channel flow events provides soil properties subsidies from upland areas; second, enhanced infiltration into wash sediment allows more locally generated rainwater to be stored below wash sediments [6, 8, 9].

### Effect of the ephemeral stream on the plant species distribution

Our study showed shrub and herbaceous species distribution differed between washes and non-washes, suggesting that ephemeral stream can affect spatial distribution of plant species in an alluvial fan of arid desert regions. It has been showed that *R*. *soongonica*, *H*. *hispidus* and *P*. *harmala* are deep-rooted plant species, whereas *S*. *kaschgaricum* and *S*. *capillata* are shallow-rooted herbaceous plants [38]. In an arid desert region, shallow-rooted plants can usually grow in a variety of environments due to their lower requirements for water and other environmental resources. Oppositely, deep-rooted plants are mostly distributed in nutrient-rich environments due to their higher requirements for soil and other environmental resources [38]. In this study, it is reasonable that ephemeral stream affected the variation in soil properties across alluvial fan in an arid desert region, and therefore deep-rooted plants have inhabited washes as compared to the shallow-rooted plants.

All tributaries on the upslope were collected into main stream at the bottom of alluvial fans (Fig 1) [2, 7]. In addition, the topography was gradually flattened from the top to the bottom of the alluvial fan [2, 7]. Under the processes of these two above natural phenomena, it is not difficult to speculate that flow velocity will gradually decrease while stream flow would increase from the top to the bottom of alluvial fans [2]. Previous studies suggested the majority of stream water is converted into groundwater during the channel flow events in alluvial fans of arid regions [2]. Thus, the rates and amounts of permeation from stream into groundwater will increase from the top to the bottom of alluvial fans, as well as will have the positive relationship with streamflow in arid desert region. In these cases, the percentages of gravel and sand, the deposition soil organic matter and nutrients would be positively related with stream flow and would increase from the top to the bottom of an alluvial fan. However, due to the features of chance and suddenness of the ephemeral stream, numerous current studies indicated that stream flow is difficult to monitor in the field environment [39, 40]. Therefore, wash width is usually used to represent the flow of the ephemeral stream. It is reasonable that stream flow has a positive relationship with wash width [39, 40]. Therefore, we showed that soil properties were superior in wider washes. These improvements in soil properties and plant growing conditions can attract more deep-rooted plants to settle down in washes of alluvial fans [6, 8, 9]. Hence, our results showed that wash width has a positive linear relationship with the total number of individuals, plant height and crown area per length of washes. This suggests that the number and concentrated degree of deep-rooted plants are higher in wider than those in narrower positions of washes. In other words, the spatial distributions of deep-rooted plants are affected by the ephemeral stream in an alluvial fan of arid desert region.

## Concluding remarks

We show that the images of UAV at 20 m and 150 m above the ground can quickly and accurately interpret the field investigation data of herbaceous and shrubs in an alluvial fans of arid desert regions, respectively, suggesting that that UAV is a promising approach for exploring plant community ecology in arid desert regions. This study suggests that species diversity, the spatial distribution of plant species, and the characteristics of plant growth, such as plant height and crown area, are significantly affected by ephemeral stream probably due to direct and indirect changes via soil properties in alluvial fans of arid desert regions. The deep-rooted plants were observed to grow in washes only, whereas shallow-rooted plants can inhabit heterogeneous alluvial fans environments. This study concludes that the coupling factors of the ephemeral stream, such as soil erosion, particle transport and sedimentation, can dramatically cause changes in soil properties and the total number of individual plants, and hence influence species diversity, plant growth characteristics and spatial distribution of plant species in alluvial fan of arid desert regions.

## Supporting information

S1 Table Soil properties and sampling data. Sampling data includes wash width (m), the numbers of individual plants (n), plant height (m) and crown area (m^2^). Species types of herbaceous and shrub communities were measured at 4 m^2^ and 20 m^2^ by using low-altitude unmanned aerial vehicle (UAV), respectively. Soil properties included soil texture (sand, silt and clay, expressed in %), soil water content (%), pH, soil organic matter (mg·kg^-1^), soil electric conductivity (mS·cm^-1^), soil bulk density (kg·m^-3^) and gravel content (mass percent; %).
(XLSX)

S1 Fig Alluvial fans and their system of drainage channels in arid desert regions. (TIF)

S2 Fig Graphical Abstract. The characteristics of the ephemeral stream, such as soil erosion, particle transport and sedimentation, change soil properties and the total number of individual plants, and as a consequence influence species diversity, plant growth and spatial distribution of plant species in alluvial fan of arid desert regions.
(TIF)
