# Supplementary figures and images for: Effects of the ephemeral stream on plant species diversity and distribution in an alluvial fan of arid desert region: An application of a low altitude UAV
Source: PLoS One. 2019 Feb 27;14(2):e0212057. doi: 10.1371/journal.pone.0212057 (PMC6392318; doi:10.1371/journal.pone.0212057)

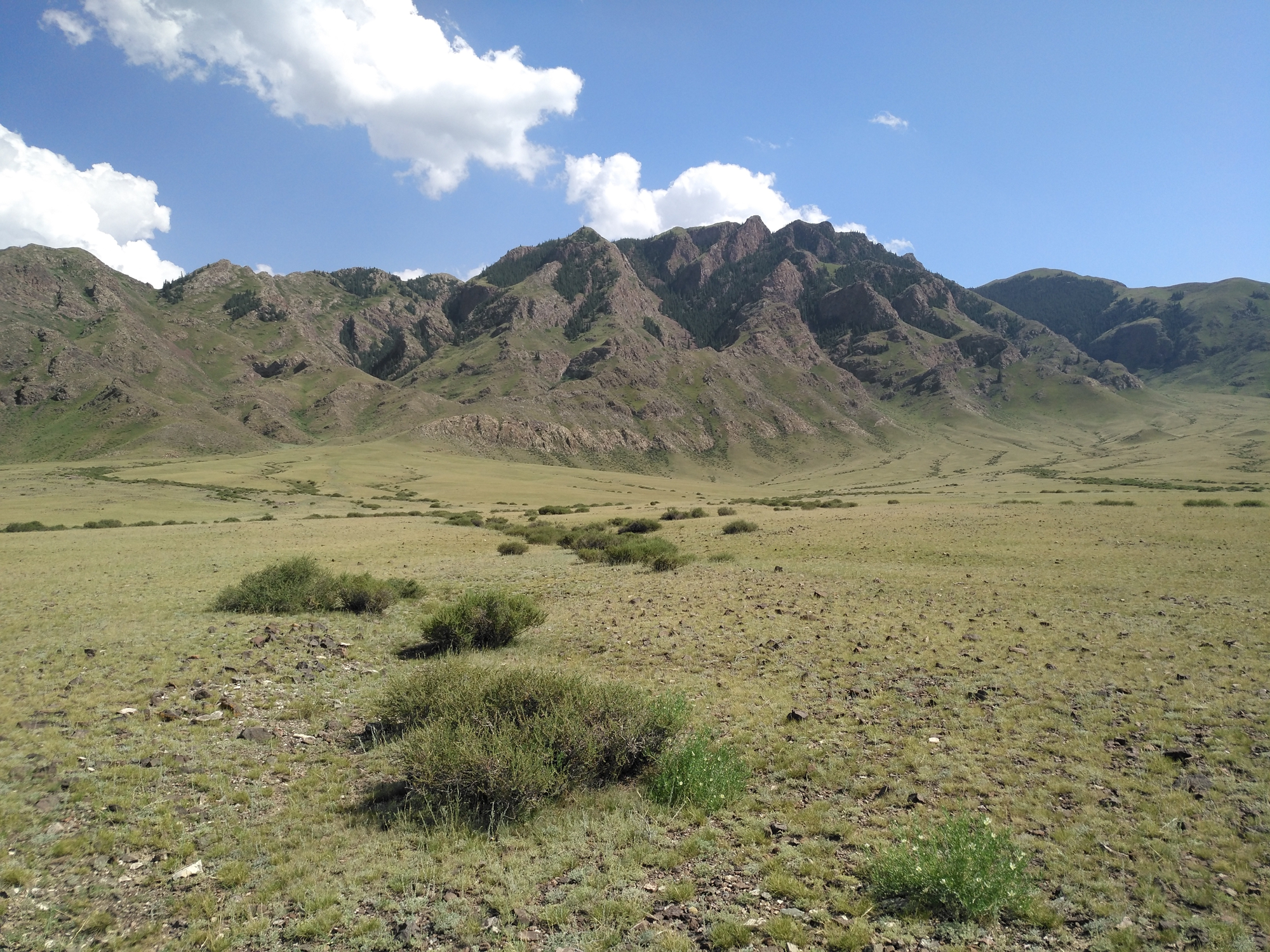

Supplement: S1 Fig — (TIF) [file pone.0212057.s002.tif]

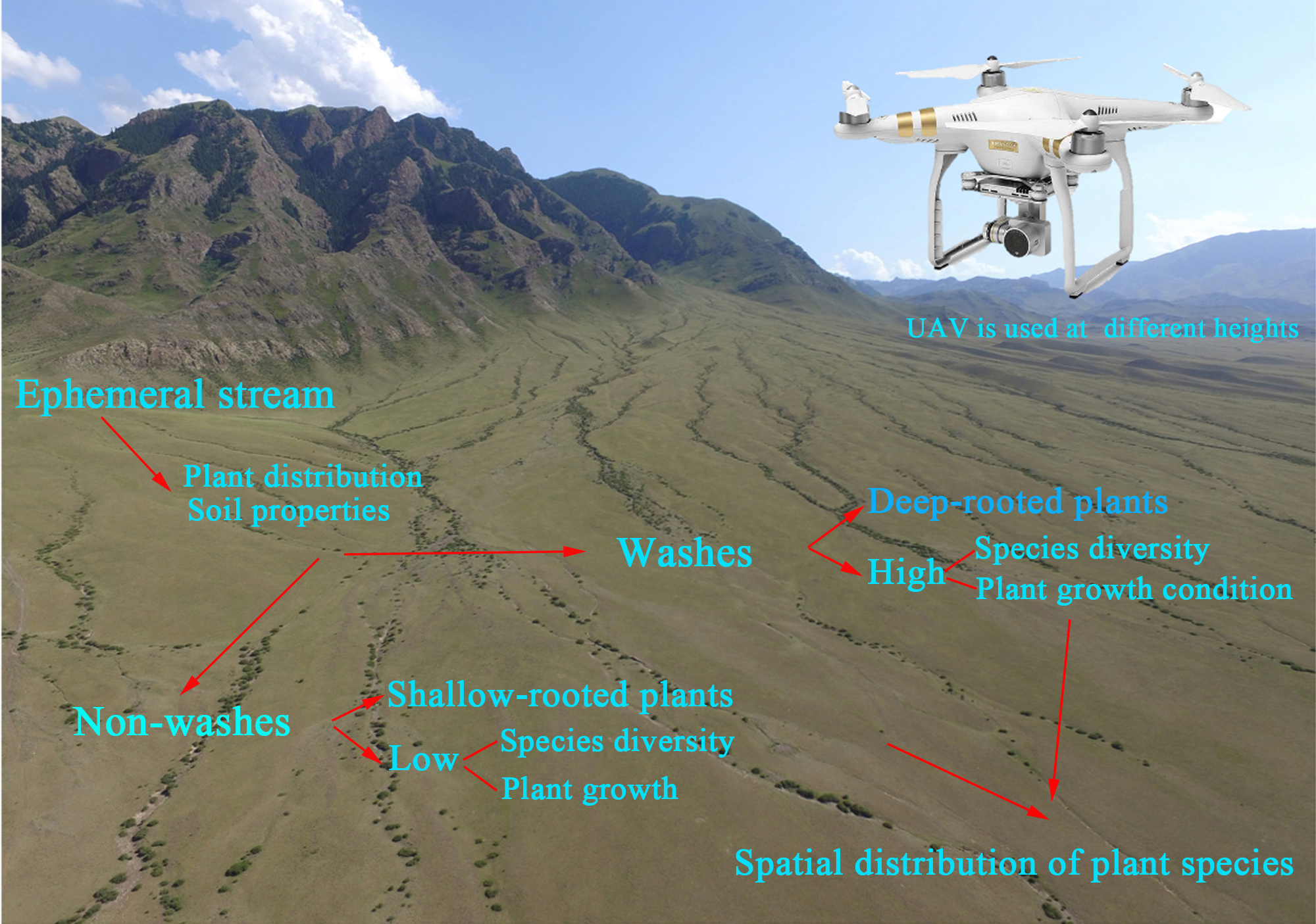

Supplement: S2 Fig — The characteristics of the ephemeral stream, such as soil erosion, particle transport and sedimentation, change soil properties and the total number of individual plants, and as a consequence influence species diversity, plant growth and spatial distribution of plant species in alluvial fan of arid desert regions. (TIF) [file pone.0212057.s003.tif]
